# Prognostic value and immunological role of FOXM1 in human solid tumors

**DOI:** 10.18632/aging.204394

**Published:** 2022-11-21

**Authors:** Guohua Wei, Xin Yang, Huangzhou Lu, Lan Zhang, Yong Wei, Hai Li, Mingxia Zhu, Xin Zhou

**Affiliations:** 1 Department of Anesthesiology, The First Affiliated Hospital of Nanjing Medical University, Nanjing 210029, China; 2 Department of Urology, The Second Affiliated Hospital of Nanjing Medical University, Nanjing 210000, China; 3 Department of Emergency, Changshu No.2 People's Hospital, The Fifth Affiliated Clinical Medical College of Yangzhou University, Changshu 215500, Jiangsu, China; 4 Department of Radiation Oncology, Shanghai Tenth People’s Hospital of Tongji University, Shanghai 200072, China; 5 Department of Pathology, The First Affiliated Hospital of Nanjing Medical University, Nanjing 210029, China; 6 Department of Radiation Oncology, The First Affiliated Hospital of Soochow University, Suzhou 215006, China; 7 Department of Oncology, The First Affiliated Hospital of Nanjing Medical University, Nanjing 210029, China

**Keywords:** FOXM1, pan-cancer, prognosis, immune infiltration, TMB

## Abstract

FOXM1 acts as an oncogenic transcription factor and is involved in multiple hallmarks of human malignancies. Recent studies have demonstrated that FOXM1 is upregulated and correlated with poor prognosis in a majority of cancers. However, there are few pan-cancer analyses of FOXM1. This study aimed to investigate the expression profiles and clinical significance of FOXM1 in 31 types of solid tumors. We explored the expression profiles and the prognostic value of FOXM1 in pan-cancer across The Cancer Genome Atlas (TCGA). We further used lung adenocarcinoma (LUAD) tissues combined with quantitative real-time PCR (qRT-PCR) and immunohistochemistry (IHC) for experimental validation of FOXM1 expression. Besides, we verified the function of FOXM1 in a lung cancer cell line. Gene set enrichment analysis (GSEA) was conducted to explore signaling pathways related to FOXM1 expression. We observed that up-regulated FOXM1 was significantly related to poor survival in most tumors. Furthermore, there are significant correlations between FOXM1 expression and the infiltrating levels of different types of immune cells, TMB, MSI and immune checkpoint genes in a variety of cancers. Additional analysis based on IMvigor 210 cohort confirmed that patients with high level of FOXM1 exhibited a superior response to anti-PD-L1 therapy, and had a prolonged OS. In conclusion, this study indicated that FOXM1 could serve as a prognostic biomarker for most types of cancers and played a crucial role in the tumor immune microenvironment.

## INTRODUCTION

FOXM1 belonging to the conserved forkhead box (FOX) transcription factor family, significantly contributes to cancer development and progression [1]. In addition to regulating cell proliferation and migration, FOXM1 also affects angiogenesis, inflammation, chemotherapy drug resistance and radiation resistance in human cancers [2, 3]. Recent research has demonstrated that FOXM1 is overexpressed in most cancers, such as bladder cancer and cholangiocarcinoma, and related to poor prognosis [4–6]. Nevertheless, there is little research on FOXM1 in pan-cancer.

The tumor microenvironment is a complicated component composed of diversified cells and extracellular matrix, and is essential for tumorigenesis and development. If the immune cells in the tumor microenvironment cannot eliminate the preneoplastic cells in time, cancer can develop and progress [7–9]. Moreover, there is increasing evidence that tumor-infiltrating immune cells significantly contribute to outcome prediction and therapeutic effectiveness [10–12]. Recently, immunotherapies, especially treatments targeting immune checkpoint inhibitors, has revolutionized cancer treatment and significantly extended overall survival of advanced cancers. Unfortunately, the benefit population is limited [13–15]. Hence, there is an urgent need to investigate tumor-immune interactions and discover potential cancer immunotherapy targets.

Here, a pan-cancer analysis of FOXM1 was conducted by the TCGA database and FOXM1 expression was verified in lung adenocarcinoma (LUAD). We also analyzed the related cell functions of FOXM1 in lung cancer cells. Furthermore, the association of FOXM1 expression with prognosis, clinical features, tumor-infiltrating immune cells, TMB, MSI, and immune checkpoint was investigated.

## RESULTS

### FOXM1 gene expression in human cancers

**Figure 1:**
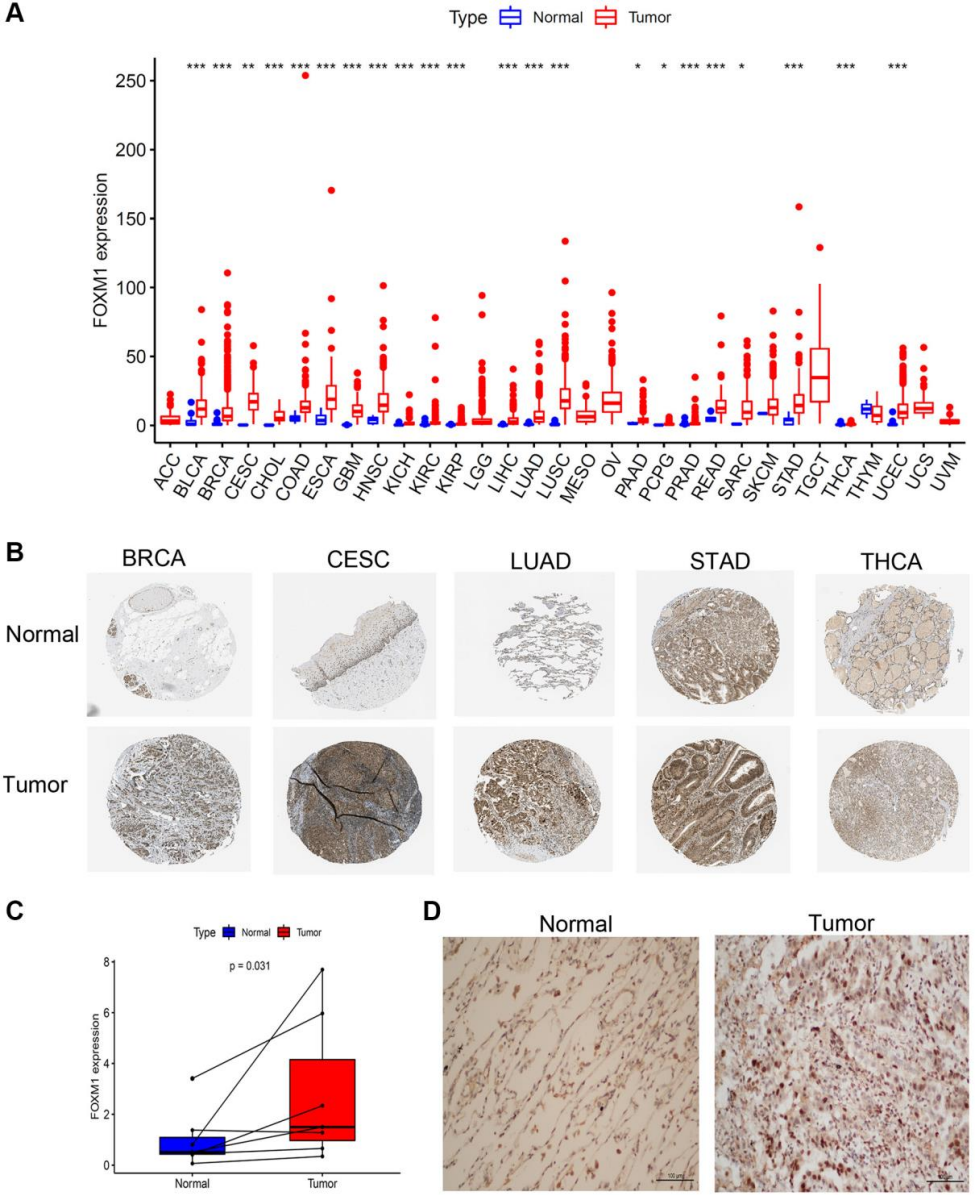
**Expression levels of FOXM1 in pan-cancer.** (**A**) Boxplot of the mRNA expression of FOXM1 in 31 types of solid tumor and normal tissues from TCGA database. (**B**) Representative IHC staining of FOXM1 in BRCA, CESC, LUAD, STAD and THCA normal and tumor tissues in HPA. (**C**) Expression of FOXM1 in tumor group were upregulated than that in normal group. (**D**) IHC analysis of FOXM1 in LUAD tissues. Representative images are shown. ^*^p < 0.05, ^**^p < 0.01, ^***^p < 0.001.

According to our findings, FOXM1 expression was significantly upregulated in 22 types of solid tumors from the TCGA database (Figure 1A). Further investigation of FOXM1 protein expression was undertaken in the HPA cohort. FOXM1 protein expression in BRCA, CESC, LUAD, STAD and THCA tumor tissues was significantly higher (Figure 1B). Next, our study validated FOXM1 mRNA expression in 7 matched LUAD normal and tissues by quantitative real-time PCR (qRT-PCR). LUAD tissues showed significantly increased FOXM1 expression levels compared with normal tissues (Figure 1C). Immunohistochemistry was further used to assess FOXM1 protein expression levels, demonstrating that LUAD had higher levels of FOXM1 protein expression than normal tissues (Figure 1D).

**Figure 2:**
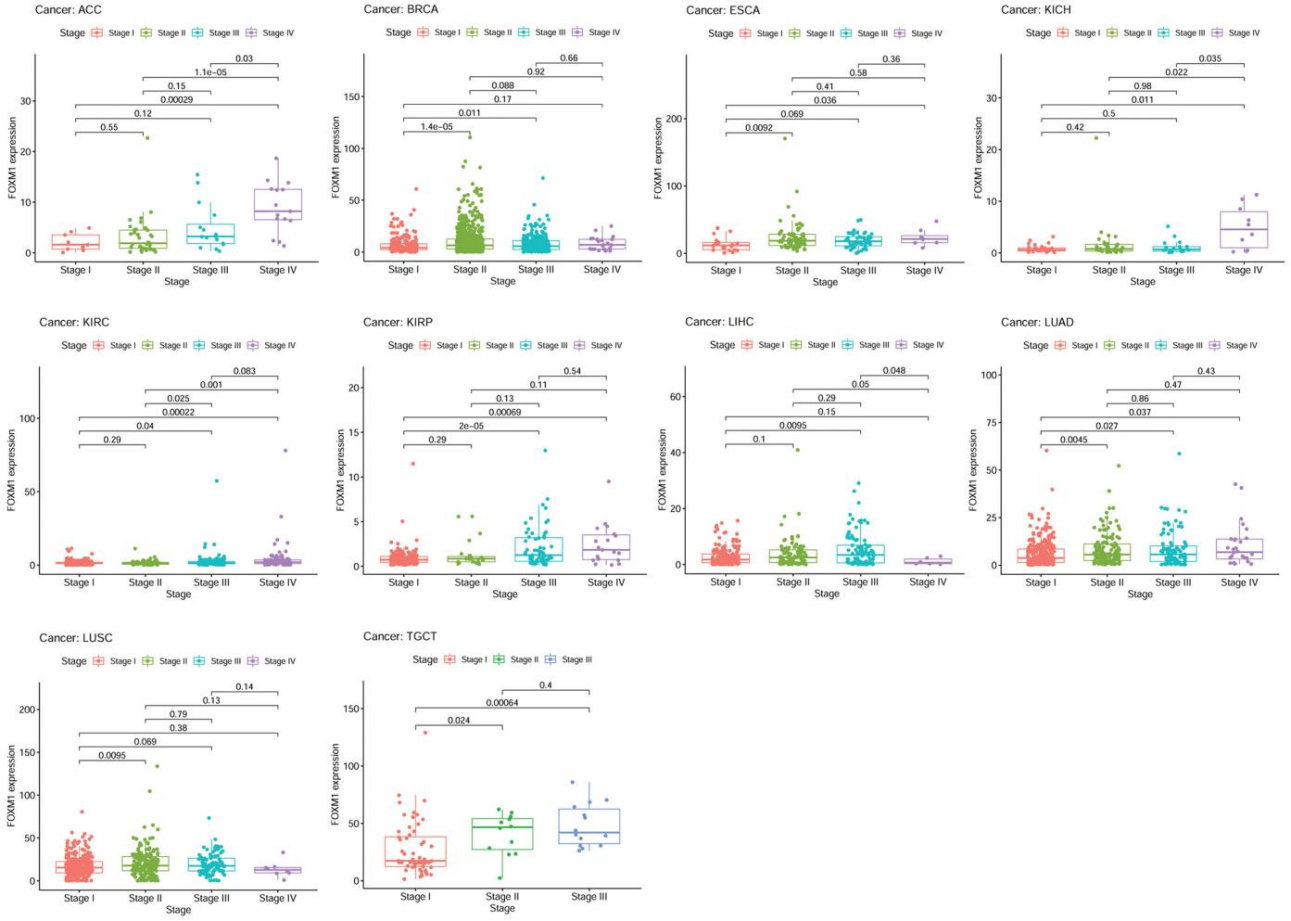
**The association of FOXM1 expression with the pathological stages of cancers.*** p* < 0.05 was considered significant.

We next evaluated FOXM1 expression according to tumor stage, gender and age. Higher FOXM1 expression was dramatically related to advanced tumor stage in ACC, BRCA, ESCA, KICH, KIRC, KIRP, LIHC, LUAD, LUSC, TGCT (Figure 2). Interestingly, ACC, ESCA, KICH, KIRC, KIRP, LUAD patients with stage IV tumors expressed more FOXM1 than patients with stage I tumors. A comparison of FOXM1 expression in tumors according to gender and age is shown in Supplementary Figure 1.

### Pan-cancer prognostic value of FOXM1

**Figure 3:**
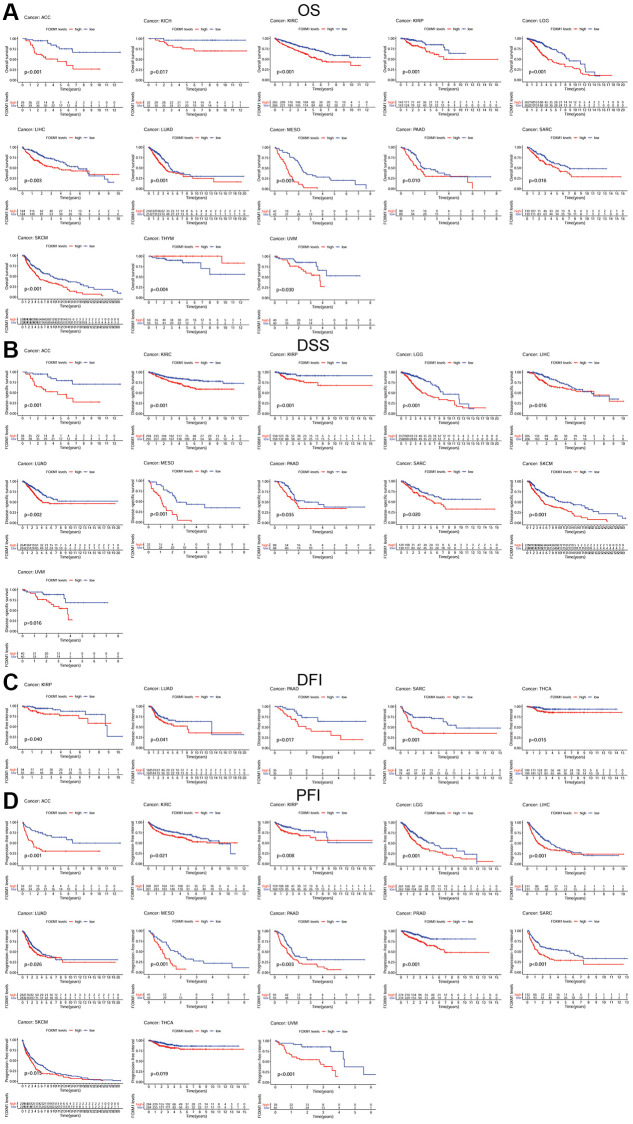
**Kaplan-Meier survival curves comparing the expression of FOXM1 in pan-cancer.** (**A**) Kaplan–Meier OS curves for the low expression group and high expression group of FOXM1 in 13 types of cancers; (**B**) Kaplan–Meier DSS curves for the low expression group and high expression group of FOXM1 in 11 types of cancers; (**C**) Kaplan–Meier DFI curves for the low expression group and high expression group of FOXM1 in 5 types of cancers; (**D**) Kaplan–Meier PFI curves for the low expression group and high expression group of FOXM1 in 13 types of cancers. *p* < 0.05 was considered significant.

To investigate how FOXM1 expression relates to prognosis, survival analyses were performed by the TCGA cohort. Patients with higher FOXM1 expression had poor overall survival (OS) in ACC, KICH, KIRC, KIRP, LGG, LIHC, LUAD, MESO, PAAD, SARC, SKCM, UVM based on Kaplan-Meier analysis. Conversely, patients with higher FOXM1 expression had better OS in THYM. In order to avoid the bias resulting from non-tumor related death, the relationship of FOXM1 expression with a poor disease-specific survival (DSS) was further evaluated. The results, much like that of the OS analysis, demonstrated that higher FOXM1 expression significantly predicted a poor DSS. There was a negative correlation between disease-free interval (DFI) and FOXM1 expression in KIRP, LUAD, PAAD, SARC, and THCA. Our study finally analyzed the association between FOXM1 expression and progression-free interval (PFI). Patients with higher FOXM1 expression had poor PFI in ACC, KIRC, KIRP, LGG, LIHC, LUAD, MESO, PAAD, PRAD, SARC, SKCM, THCA, UVM (Figure 3).

**Figure 4:**
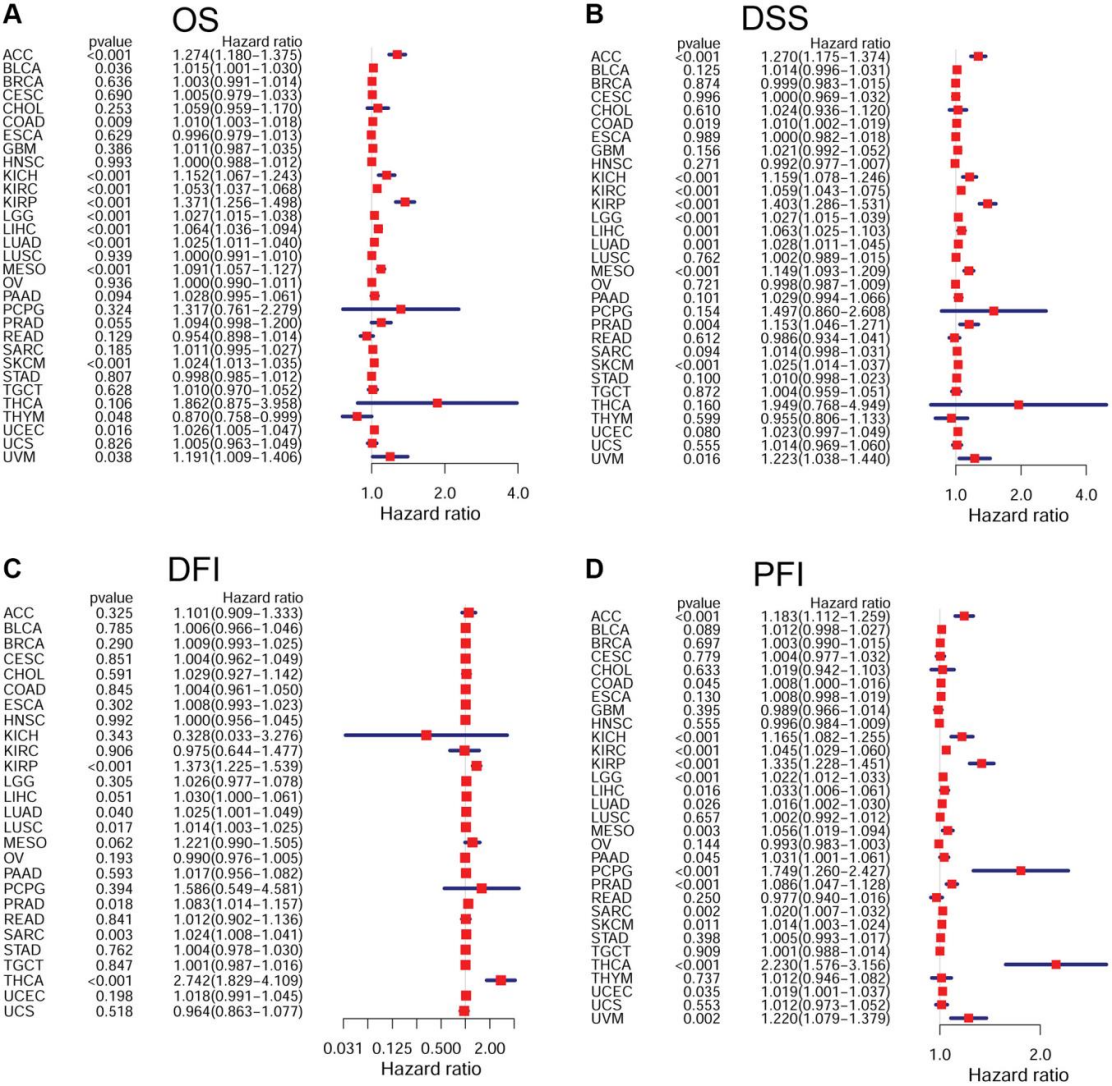
**Forest plots of Cox regression analyses in pan-cancer.** (**A**) Association of FOXM1 expression with OS; (**B**) Association of FOXM1 expression with DSS; (**C**) Association of FOXM1 expression with DFI; (**D**) Association of FOXM1 expression with PFI. *p* < 0.05 was considered significant.

According to the forest plots, FOXM1 could significantly affect the OS of 13 types of solid tumors. The expression of FOXM1 represented a high-risk indicator in ACC, BLCA, COAD, KICH, KIRC, KIRP, LGG, LIHC, LUAD, MESO, SKCM, UCEC, UVM, but a low-risk indicator in THYM. For DSS, similar results were observed. Regarding FOXM1 and DFI, Cox regression analysis revealed that FOXM1 expression impacted the DFI of KIRP, LUAD, LUSC, PRAD, SARC, THCA. In terms of PFS, forest plot revealed that the hazard ratios for FOXM1 were significant for 18 cancer types (Figure 4).

### Association of FOXM1 with tumor immune microenvironment

**Figure 5:**
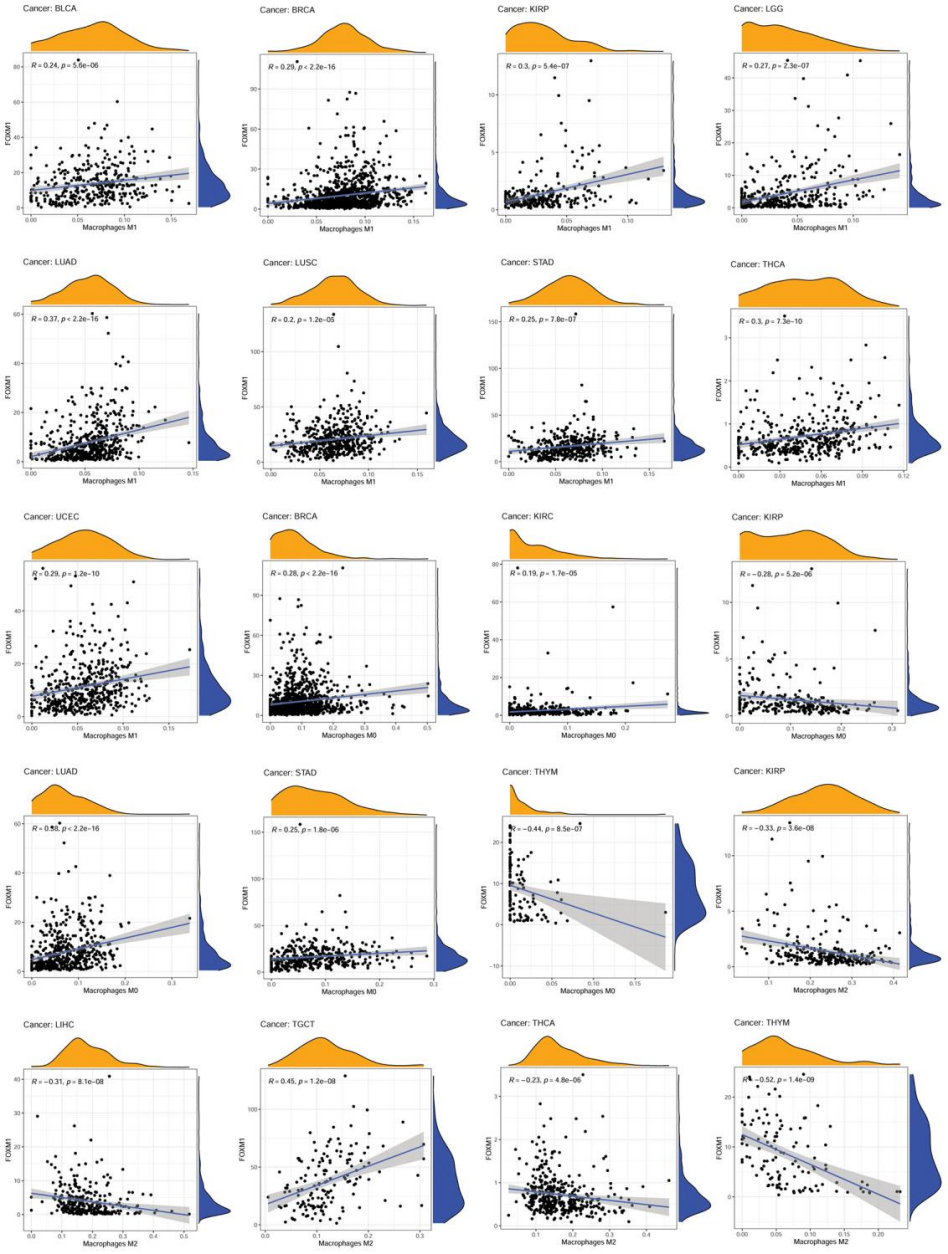
**Association of FOXM1 expression with macrophages.*** p* < 0.0001 was considered significant.

In order to understand how FOXM1 expression correlates with immune infiltration, we used the Estimating Relative Subsets of RNA Transcripts (CIBERSORT) method. Different subpopulations of invasive macrophages showed a correlation with FOXM1 expression. There was a positive association of FOXM1 expression with macrophage M1 in 9 types of cancers. BRCA, KIRC, LUAD, and STAD revealed a positive association of FOXM1 expression with macrophage M0, whereas KIRP and THYM showed a negative correlation. A positive correlation was found between FOXM1 expression and macrophage M2 infiltration in TGCT and THYM, but a negative correlation was found in KIRP, LIHC, and THCA (Figure 5). Moreover, there was a correlation between FOXM1 expression and infiltration levels of CD4+T cells, B cells, CD8+T cells, mast cells, dendritic cells, T cells follicular helper, NK cells and Tregs (Supplementary Figure 2).

**Figure 6:**
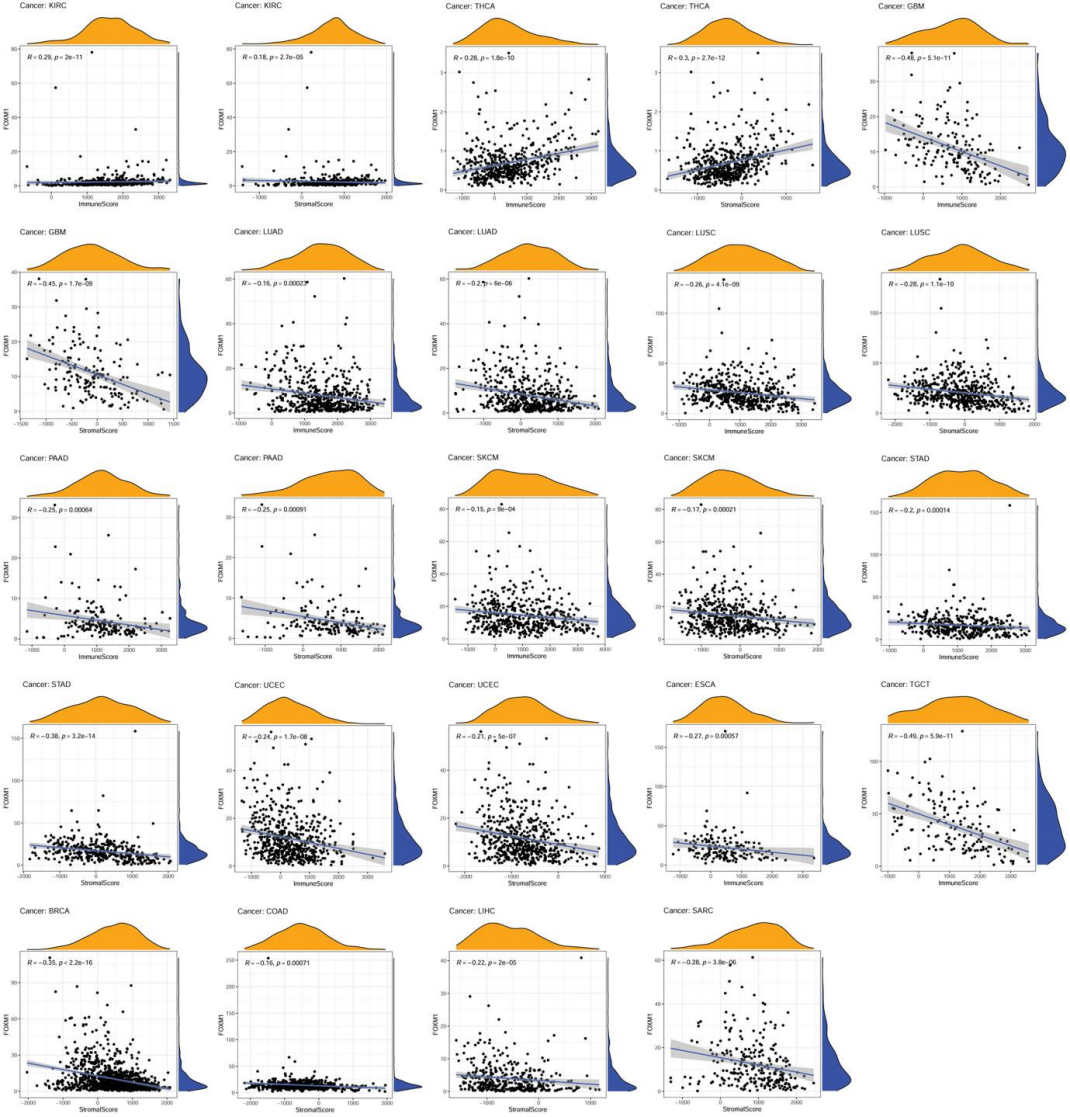
**Association of FOXM1 expression with the immune score and stomal score.*** p* < 0.05 was considered significant. *p* < 0.001 was considered significant.

Next, it was explored whether the expression of FOXM1 and immune scores correlated using the ESTIMATE algorithm. FOXM1 expression was positively associated with immune and stromal scores of KIRC and THCA, but negatively associated with GBM, LUAD, LUSC, PAAD, SKCM, STAD, and UCEC. Additionally, a negative relationship was found between FOXM1 expression and the ESCA and TGCT immune scores, and a negative relationship was found between FOXM1 expression and the BRCA, COAD, LIHC, and SARC stromal scores (Figure 6).

### Association of FOXM1 expression with TMB and MSI

**Figure 7:**
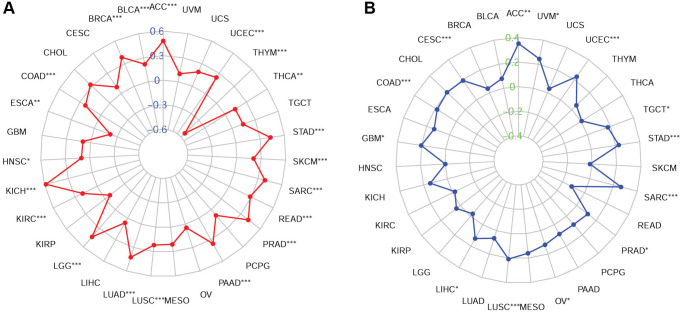
**Associations of FOXM1 expression with TMB and MSI in cancers.** (**A**) Radar map of association of FOXM1 expression with TMB, (**B**) Radar map of association of FOXM1 expression with MSI. ^*^p < 0.05, ^**^p < 0.01, ^***^p < 0.001.

We further explored the association of TMB and MSI with FOXM1 expression. A positive correlation was found between FOXM1 and TMB in 19 cancer types. While in ESCA and THYM, negative relationships were found (Figure 7A). Additionally, FOXM1 and MSI had a positive correlation in 13 cancer types, including ACC, CESC, CHOL, COAD, GBM, LIHC, LUSC, OV, PAAD, SARC, STAD, TGCT, UCEC, UVM (Figure 7B).

### Correlations of FOXM1 with immune checkpoint-associated genes

**Figure 8:**
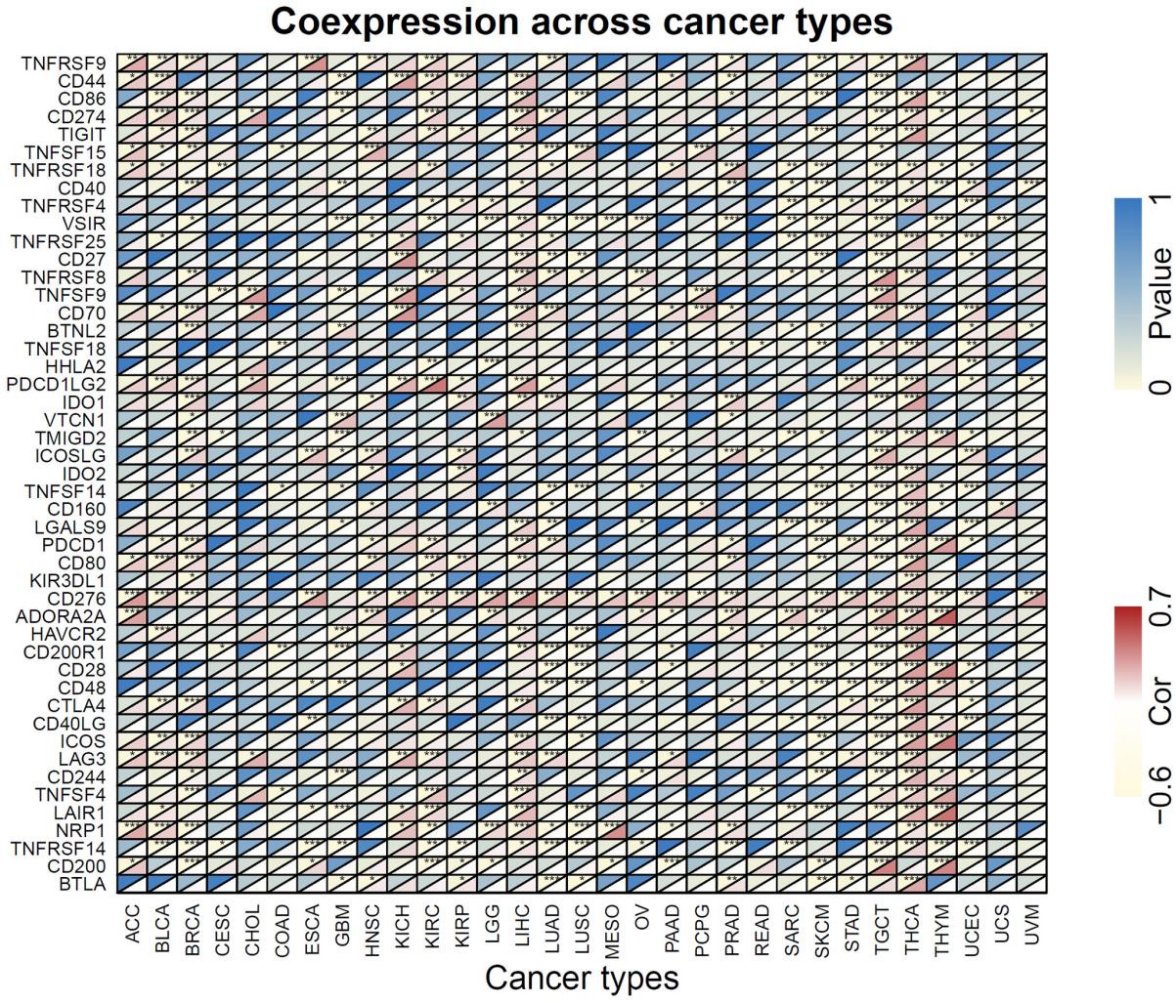
**Heatmap of the association of FOXM1 expression with 47 common immune checkpoints gene levels in 31 types of cancers.** For each pair, the top left triangle represents the *P*-value, and the bottom right triangle represents the correlation coefficient. ^*^p < 0.05, ^**^p < 0.01, ^***^p < 0.001.

A gene coexpression analysis was conducted to investigate how FOXM1 is related to immune checkpoint genes. More than 40 immune checkpoint genes were assessed. In LIHC, SKCM, and THCA, FOXM1 expression correlated significantly with the major checkpoint genes. Interestingly, CD276 was positively associated with FOXM1 expression in most cancers, with the exception of CESC, CHOL, COAD, GBM, READ, SARC and UCS. Forty-one immune checkpoint markers had significant associations with FOXM1 expression in TGCT. Furthermore, CESC, CHOL, COAD, READ, USC, and UVM showed relatively small correlations (Figure 8).

### Immunotherapeutic response prediction value of FOXM1

**Figure 9:**
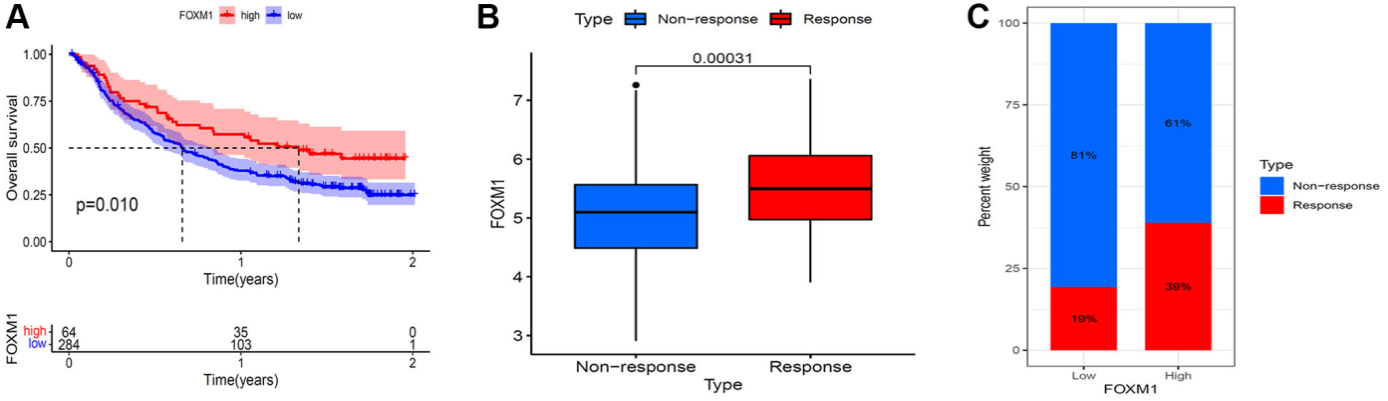
**Validation of the immunotherapeutic predictive value of FOXM1.** (**A**) Kaplan–Meier OS curves for patients with FOXM1 high and low expression subgroups in IMvigor 210 cohort. (**B**) FOXM1 expression was higher in responders than that in non-responders. (**C**) The proportion of patients with response to anti- PD-L1 therapy in FOXM1 high and low expression groups. *p* < 0.05 was considered significant.

As FOXM1 was closely related with tumor immune microenvironment, we sought to confirm whether it could predict response to immune checkpoint inhibitor treatment based on IMvigor 210 cohort. In patients with high FOXM1 levels, OS was significantly longer than in those with low levels (Figure 9A). Furthermore, higher FOXM1 was tested in responders than that in non-responders (Figure 9B). Anti-PD-L1 therapy was significantly more effective when FOXM1 levels were higher. (Figure 9C).

### Gene set enrichment analysis

Kyoto Encyclopedia of Genes and Genomes (KEGG) and Gene Ontology (GO) pathways enrichment was analyzed to investigate the biological significance of FOXM1 expression in various cancers (Supplementary Figures 3 and 4). In KEGG analysis, FOXM1 was related to “cell cycle” and “DNA replication” pathways in multiple tumors. Besides, FOXM1 could modulate some pathways, such as “cytokine receptor interactio”, “complement and coagulation cascades”. In GO analysis, FOXM1 could modulate some pathways, such as “antigen binding”, “B cell mediated immunity”, “T cell receptor complex” in CHOL; “adaptive immune response based on somatic recombination”, “immunoglobulin production” in KIRC; “antigen binding”, “Fc receptor mediated stimulatory”, “humoral immune response circulating immunoglobulin”, “negative regulation of immune response”, “positive T cell selection” in TGCT; “antigen binding”, “humoral immune response”, “immunoglobulin complex” in UCS. FOXM1 also regulated many other pathways associated with “cell cycle” and “chromosome segregation”. These results demonstrated that FOXM1 widely regulated cell proliferation and immunity signaling pathways.

### Functional analysis of FOXM1 in lung cancer cells

**Figure 10:**
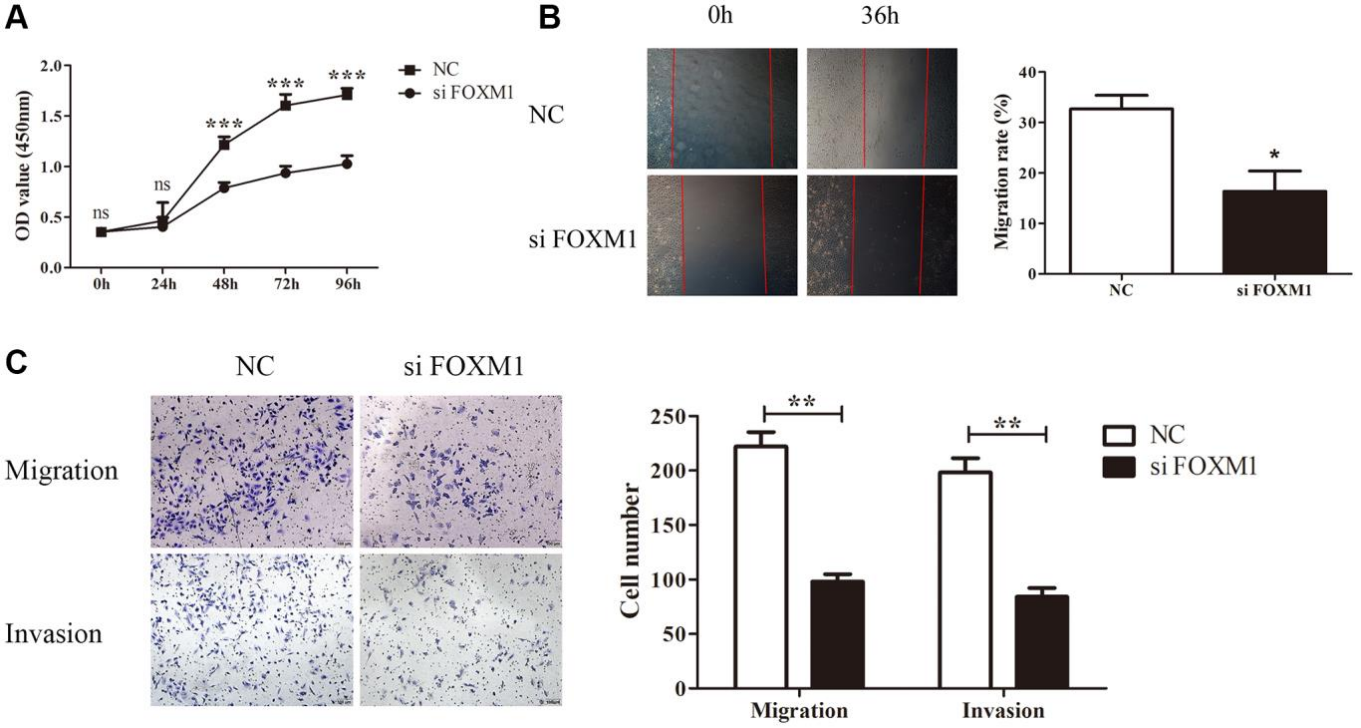
**FOXM1 knockdown inhibits lung cancer cell proliferation, migration and invasion.** (**A**) The proliferation curves of A549 cells transfected with si-NC and si- FOXM1. (**B**) The wound healing assay results for A549 cells transfected with si-NC and si-FOXM1 at 0, 36 h, respectively. (**C**) The transwell migration and invasion assay for A549 cells transfected with si-NC and si-FOXM1. ^*^p < 0.05, ^**^p < 0.01, ^***^p < 0.001.

We additionally selected a lung cancer cell line A549 as an example and performed functional experiments to further verify our bioinformatics findings. We designed and transfected siRNA of FOXM1 to knock down endogenous FOXM1 expression. The results of CCK8 assays identified that knockdown of FOXM1 with siRNA resulted in the decrease of the proliferation ability of A549 cells (Figure 10A). According to wound healing test, FOXM1 downregulation markedly diminished cell migration of A549 cells (Figure 10B). Similarly, transwell migration analysis confirmed that FOXM1 downregulation could inhibit A549 cells migration. Transwell invasion analysis also showed that after knockdown of FOXM1, A549 cells revealed a decrease in invasion capacity (Figure 10C).

## DISCUSSION

The oncogene FOXM1 plays a crucial role in a wide range of malignancies in humans [2, 3]. In addition, FOXM1 can predict poor prognosis in some cancers [4–6]. More studies focus on pan-cancer analyses of oncogenes, such as HILPDA [16], LCN2 [17], and SND1 [18] etc. These studies were performed on the basis of the publicly funded TCGA project or the accessible GEO database, which provided us with a convenience to carry out pan-cancer analysis. Our study examined FOXM1 expression profiles and prognostic significance across 31 solid tumors. In addition, FOXM1’s potential role in tumor immunology was investigated in different cancers.

We observed that, compared with normal tissues, FOXM1 was abnormally overexpressed in most tumors. Besides, FOXM1 expression significantly correlated with clinical stage, age and gender. In stage I and IV tumors, FOXM1 expression differed significantly. FOXM1 has proven to be an effective prognostic biomarker for most cancers. Furthermore, FOXM1 expression in LUAD was validated using qRT- PCR and immunohistochemistry. In line with bioinformatic analysis, in tumor tissues, significantly higher levels of FOXM1 mRNA and protein were detected.

Cox regression and Kaplan-Meier analyses revealed that most tumor types had poor prognosis when FOXM1 expression increased, especially KIRP and LUAD. These discoveries consisted with previous studies that reported FOXM1 as a negative prognostic biomarker [4–6]. However, our study was the first to demonstrate that FOXM1 upregulation was related to better OS in THYM, though the limited sample size might have contributed to this finding. Due to the consistent results in mRNA and protein expression analysis and the coincident effect of FOXM1 on clinical outcomes, we selected LUAD as an example to verify our bioinformatics findings in the manuscript. In addition, we performed functional studies on LUAD cell A549 to preliminarily evaluate the role of FOXM1 in lung cancer. The knockdown of FOXM1 effectively inhibited A549 cells proliferation, migration, and invasion. Our results were in line with the bioinformatics analysis and expression analysis of FOXM1 in LUAD tissue, thus demonstrating the reliability of our study.

Therapy targeting immune checkpoint genes has been widely proven to be a successful strategy for most advanced cancers. However, some cancer types respond well to treatment only in a limited proportion of patients [19]. Thus, in order to predict the outcome of immune checkpoint inhibitor therapy, reliable biomarkers must be identified.

TMB represents the cumulative number of somatic non-synonymous mutations per Mb in the genomic sequence. High TMB can produce more neoantigens and make the tumor more immunogenic, thus, enhancing T cell response and anti-tumor response [20–22]. TMB has been well known to act as a biomarker of immune checkpoint inhibitor response. Recent studies have reported that patients with high TMB tend to benefit from immunotherapy and have better response and OS [23, 24].

MSI-H reflects the change in microsatellite length between normal and tumor cells caused by repeated unit insertions or deletions. MSI-H was shown to be caused by mismatch-repair deficiencies (dMMR) and was an important predictor of tumor development [25]. Both MSI-H and dMMR were associated with high mutation load, which was more likely to benefit from immunotherapy. Recent reports have also indicated that anti-PD-1 immunotherapy is greatly effective against MSI-H solid tumors [26–30]. Pembrolizumab has received FDA approval for treating MSI-H/dMMR solid tumors [31]. In our study, FOXM1 expression was found to have an impact on TMB and MSI, thereby affecting immunotherapy response.

Tumor infiltrating lymphocytes are crucial for tumor immunity in inhibiting or promoting tumor progression [32]. A recent finding indicated that the status of sentinel lymph nodes and the survival of patients could be predicted independently using tumor infiltrating lymphocytes [33]. Tumor-infiltrating lymphocytes could also affect the clinical efficacy of immunotherapy [34, 35]. PD-1/PD-L1 inhibitor therapy might be more effective for various types of cancer when CD8+ T cells were more abundant in the tumor core as well as at the invasive margin [36, 37]. In melanoma tumors that responded to anti-CTLA-4, increased numbers of several subsets of CD4+Th1 cells have been observed [38]. The role of B cells in immune checkpoint inhibitors has also been demonstrated in recent studies [39, 40]. Moreover, tumor-associated macrophages (TAMs) influenced immune checkpoint inhibitors response in addition to contributing to tumor progression. For instance, in NSCLC, there was an increase in M2 macrophage infiltration in patients with hyperprogression under PD-1/PD-L1 inhibitor therapy [41]. The infiltration of CD68+CD16+ classically activated M1 macrophages was higher in melanoma tumors that responded to anti-CTLA-4 [42]. In this study, the expression of FOXM1 and multiple immune cell subtypes infiltration correlated significantly. Moreover, FOXM1 expression and immune checkpoint gene correlated significantly. The analysis of immunotherapy cohort (IMvigor 210) showed that patients with higher FOXM1 expression responded better to anti-PD-L1 treatment, and had a longer OS. Our findings suggested that FOXM1 played a vital role in tumor immunology.

In conclusion, our study demonstrated that multiple tumors that overexpressed FOXM1 had poor prognoses. Furthermore, immune cell infiltration, immune checkpoint genes, TMB, and MSI were associated with aberrant FOXM1 expression. However, this study mainly focused on bioinformatics and public databases. In order to fully understand how FOXM1 affects tumor progression and immunity, further mechanistic studies are needed. Therefore, there is potential for FOXM1 to be used as a biomarker for cancer prognosis and immunotherapy.

## METHODS

### Data collection

RNA sequencing datasets, clinicopathological information and survival information for 31 solid tumors were acquired from the TCGA database (https://portal.gdc.cancer.gov/) on March 20, 2021. Table 1 provides a general overview of pan-cancer. Patient transcriptomic and clinical data for BLCA receiving anti-PD-L1 therapy (atezolizumab) were obtained from the IMvigor 210 trial data [43]. R version 3.6.3 was used for all data analysis.

**Table 1:** Pan-cancer data acquired from TCGA.

**Cancer type**	**Full name**	**Tumor samples**	**Normal samples**
ACC	Adrenocortical carcinoma	79	0
BLCA	Bladder urothelial carcinoma	414	19
BRCA	Breast invasive carcinoma	1109	120
CESC	Cervical squamous cell carcinoma and endocervical adenocarcinoma	306	3
CHOL	Cholangiocarcinoma	36	9
COAD	Colon adenocarcinoma	480	41
ESCA	Esophageal carcinoma	162	11
GBM	Glioblastoma multiforme	169	5
HNSC	Head and neck squamous cell carcinoma	502	44
KICH	Kidney chromophobe	65	24
KIRC	Kidney renal clear cell carcinoma	539	72
KIRP	Kidney renal papillary cell carcinoma	289	32
LGG	Brain lower grade glioma	529	0
LIHC	Liver hepatocellular carcinoma	374	50
LUAD	Lung adenocarcinoma	535	59
LUSC	Lung squamous cell carcinoma	502	49
MESO	Mesothelioma	86	0
OV	Ovarian serous cystadenocarcinoma	379	0
PAAD	Pancreatic adenocarcinoma	178	4
PCPG	Pheochromocytoma and paraganglioma	183	3
PRAD	Prostate adenocarcinoma	499	52
READ	Rectum adenocarcinoma	167	10
SARC	Sarcoma	263	2
SKCM	Skin cutaneous melanoma	471	1
STAD	Stomach adenocarcinoma	375	32
TGCT	Testicular germ cell tumors	156	0
THCA	Thyroid carcinoma	510	58
THYM	Thymoma	119	2
UCEC	Uterine corpus endometrial carcinoma	552	35
UCS	Uterine carcinosarcoma	56	0
UVM	Uveal melanoma	80	0

### FOXM1 expression analysis

Using the Wilcoxon test, we compared FOXM1 expression levels in tumor and normal tissue. Then, a box plot was displayed by the ‘ggpubr’ R package. From the HPA database, immunohistochemistry images of the FOXM1 protein were obtained for both normal and cancerous tissues. We also explored differences in FOXM1 expression across different stages, genders and ages using chi-square or Fisher’s exact tests. It is statistically significant if *p* < 0.05.

### Isolation of RNA and qRT-PCR analysis

The First Affiliated Hospital of Nanjing Medical University provided the LUAD tissue. TRIzol reagent (Invitrogen, USA) was applied to extract total RNA, and PrimeScript RT reagent Kit (Takara Bio) was used to reversely transcribe into cDNA. For the detection of FOXM1 mRNA expression levels, qRT-PCR was conducted with an ABI StepOnePlus system (Applied Biosystems) and Sybrgreen-based qRT-PCR assays. GAPDH was used for normalizing the samples. Using 2^−ΔΔCt^ method, relative expression levels of mRNA were determined and each sample was performed in triplicate. The primers: FOXM1 (forward: 5′-TCTGCCAATGGCAAGGTCTCCT-3′ and reverse: 5′-CTGGATTCGGTCGTTTCTGCTG-3′).

### Immunohistochemistry

The First Affiliated Hospital of Nanjing Medical University provided Archival formalin fixed paraffin-embedded (FFPE) specimens of LUAD tissues and corresponding para-tumor tissues. All of the tumor samples were again independently confirmed by two pathologists. Thin sections (5 mm) were cut from FFPE biopsy specimens. Following deparaffinization, the sample was rehydrated with gradient alcohol. The antigens were repaired by microwaving with citrate buffer followed by incubation with 3% H_2_O_2_. A primary antibody for FOXM1 (catalog no., bs-21487R; 1:200 dilution; USA) was incubated overnight at 4°C, followed by a secondary antibody at 32°C for 30 minutes. Afterwards, diaminobenzidine chromogen was used to stain. In the final step, hematoxylin counterstaining was followed by dehydration and cover-slipping with permanent media.

### Survival analysis

Survival data from the TCGA was downloaded to evaluate the prognostic value of FOXM1. Based on the median FOXM1 expression, we divided each tumor sample into two groups. A Kaplan-Meier method was performed by the R packages ‘survival’ and ‘survminer’ to determine the correlation of FOXM1 with survival. Hazard ratios with 95% confidence intervals were collected by Cox proportional hazard regression analysis in 31 cancer types.

### Immune correlation analysis

CIBERSORT helps distinguish 22 tumor infiltrating immune cells from other cells in tissues [44]. The associations of FOXM1 expression with 22 immune cell subtypes were estimated by the CIBERSORT algorithm.

ESTIMATE is a tool that predicts tumor purity [45]. Stromal and immune scores were obtained using R packages ‘estimate’ and ‘limma’. The correlation between FOXM1 and these scores was determined using Spearman correlation analysis.

TMB and MSI have been found to have close links with the immune response in recent studies [20, 25]. The association of TMB/MSI with FOXM1 expression was evaluated using Spearman correlation analysis. The results were shown by the R ‘fmsb’ package in radar chart. The association of FOXM1 expression with common immune checkpoint genes was further evaluated using Pearson’s correlation analysis. The results were shown by ‘Reshape 2’ and ‘R Color Brewer’ packages in heatmaps.

### Gene set enrichment analysis

GSEA was conducted with ‘cluster-Profiler’ R package to explore the relevant signaling pathways between FOXM1 high and low expression groups in GO and KEGG. The enrichment significance criteria were |NES|>1, NOM *p* < 0.05, together with FDR *q* < 0.25.

### Cell culture and siRNA transfection

The National Institute of Cells (Shanghai, China) provided lung cancer cell line A549 for this study. RPMI1640 medium with 10% fetal calf serum (Gibco) was used to culture the cells, which were maintained at 37°C with 5% CO_2_ in a humidified atmosphere. The siRNA targeting FOXM1 were acquired from ShanghaGenePharma Co., Ltd., (Shanghai, China): Sense, 5′-GCCAAUCGUUCUCUGACAGAATT-3′ and antisense, 5′-UUCUGUCAGAGAACGAUUGGCTT-3′. A negative control siRNA (si-NC) was also obtained: Sense, 5′-UUCUCCGAACGUGUCACGUTT-3′ and antisense, 5′-ACGUGACACGUUCGGAGAATT-3′. FOXM1 siRNA or si-NC was transfected into A549 cells with Invitrogen™ Lipofectamine 2000 (Thermo Fisher Scientific, Inc., USA) as directed by the manufacturer.

### Cell proliferation assay

The proliferation of A549 cells was examined by the CCK8 assay kit, as instructed by the manufacturer. In 96-well plates, transfected A549 cells were planted at a density of 2000 cells/well. Absorbance value of each well at 450 nm was detected.

### Wound healing assay

We cultured transfected cells in a monolayer until confluence was achieved. A 200 μl pipette tip was applied to make an incision-like gap. The wound area was acquired with a microscope at 0 and 36 h after wounding. Analyzing the cell migration data was done with Image-Pro Plus 7.0 software.

### Transwell migration and invasion assay

Using a transwell chamber system, migration and invasion were assessed. For the migration assay, 24 hours after transfection, 10,000 cells containing medium supplemented with 2% serum were planted onto the upper chamber. For the invasion assay, a ratio of 1:4 was used for mixing matrigel and serum-free medium, followed by seeding the mixture into an upper chamber. Next, 10,000 cells were placed in the upper chamber. Each chamber was placed into a complete medium in both assays. When 24 hours had passed, using 95% ethanol, we fixed the invaded cells on the lower chamber surface for 30 minutes. Next, crystal violet at a concentration of 0.1% was applied to stain the cells for about 15 minutes. Under a microscope, three random fields of stained cells were observed and counted.

### Statistical analysis

Statistical analysis of the *in vitro* A549 cell assays was conducted by SPSS 20.0 software. The mean ± standard deviation (SD) of at least three experiments were presented. We defined statistically significant differences as two-sided *p* < 0.05 for Student’s *t*-tests.

## Supplementary Materials

Supplementary Figures
