# Supplementary material for: Prognostic value and immunological role of FOXM1 in human solid tumors
Source: Aging (Albany NY). 2022 Nov 21;14(22):9128–48. doi: 10.18632/aging.204394 (PMC9740373; doi:10.18632/aging.204394)
Supplement: Supplementary Figures [file aging-14-204394-s001.pdf]

SUPPLEMENTARY FIGURES

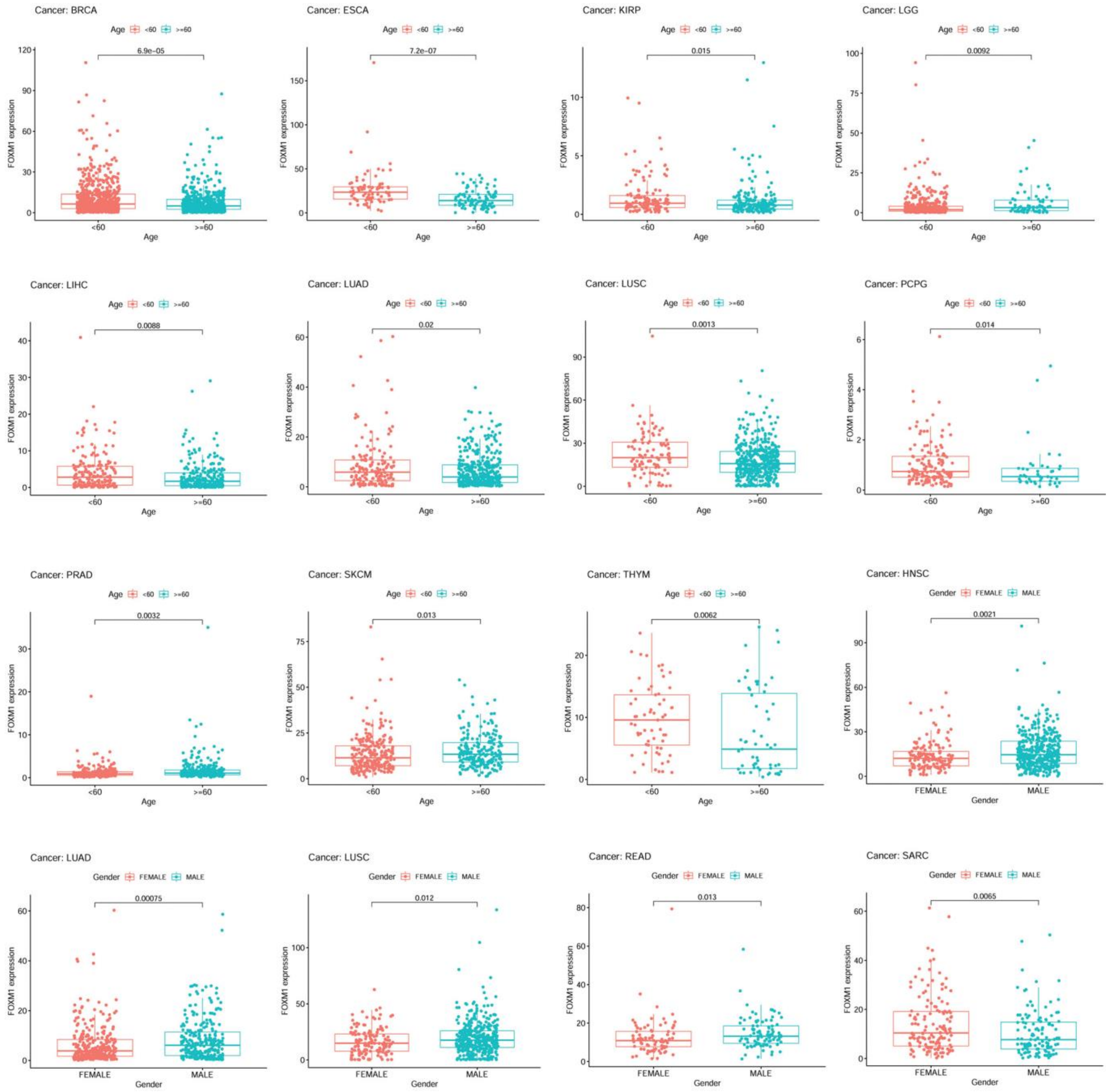

Supplementary Figure 1. Association of FOXM1 expression with patients' age and gender in pan-cancer. $p < 0.05$ was considered significant.

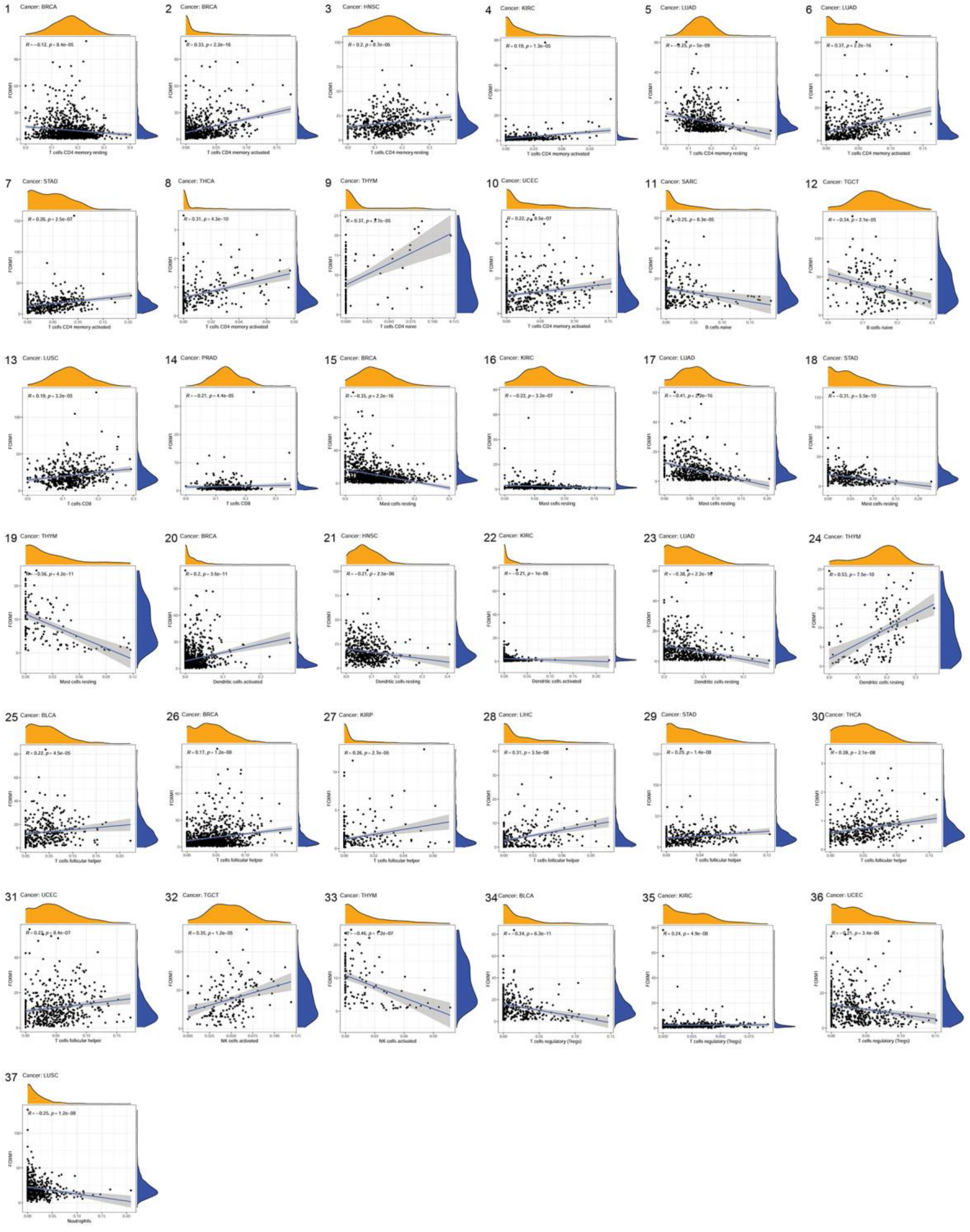

Supplementary Figure 2. Association of FOXM1 expression with immune cell infiltration levels in pan-cancer. (1–10): Association of FOXM1 expression with CD4+T cells; (11–12): Association of FOXM1 expression with B cells; (13–14): Association of FOXM1 expression with CD8+T cells; (15–19): Association of FOXM1 expression with mast cells; (20–24): Association of FOXM1 expression with dendritic cells; (25–31): Association of FOXM1 expression with T cells follicular helper cells; (32–33): Association of FOXM1 expression with NK cells; (34–36): Association of FOXM1 expression with Tregs cells; (37): Association of FOXM1 expression with neutrophils cells.

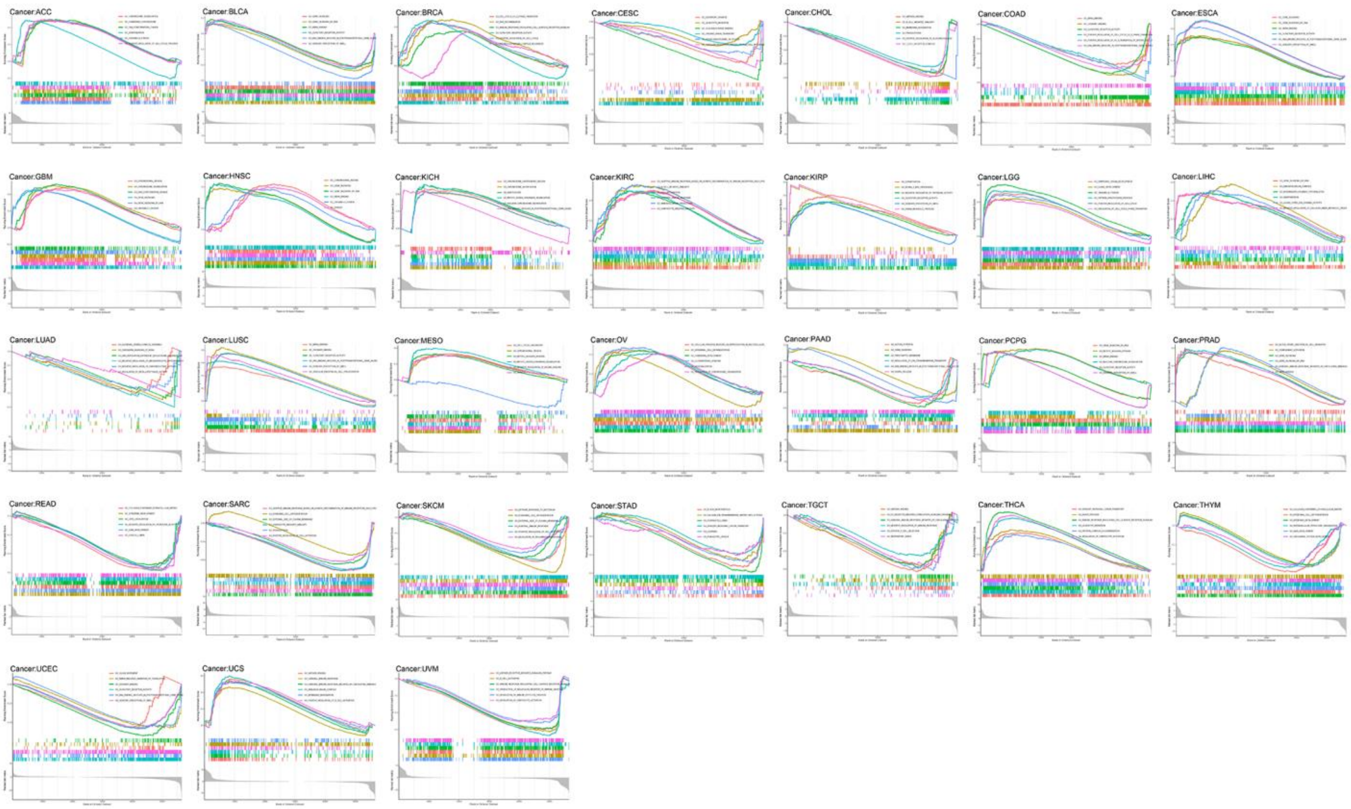

Supplementary Figure 3. Results of GSEA for FOXM1 correlation with signaling pathways in GO collection.

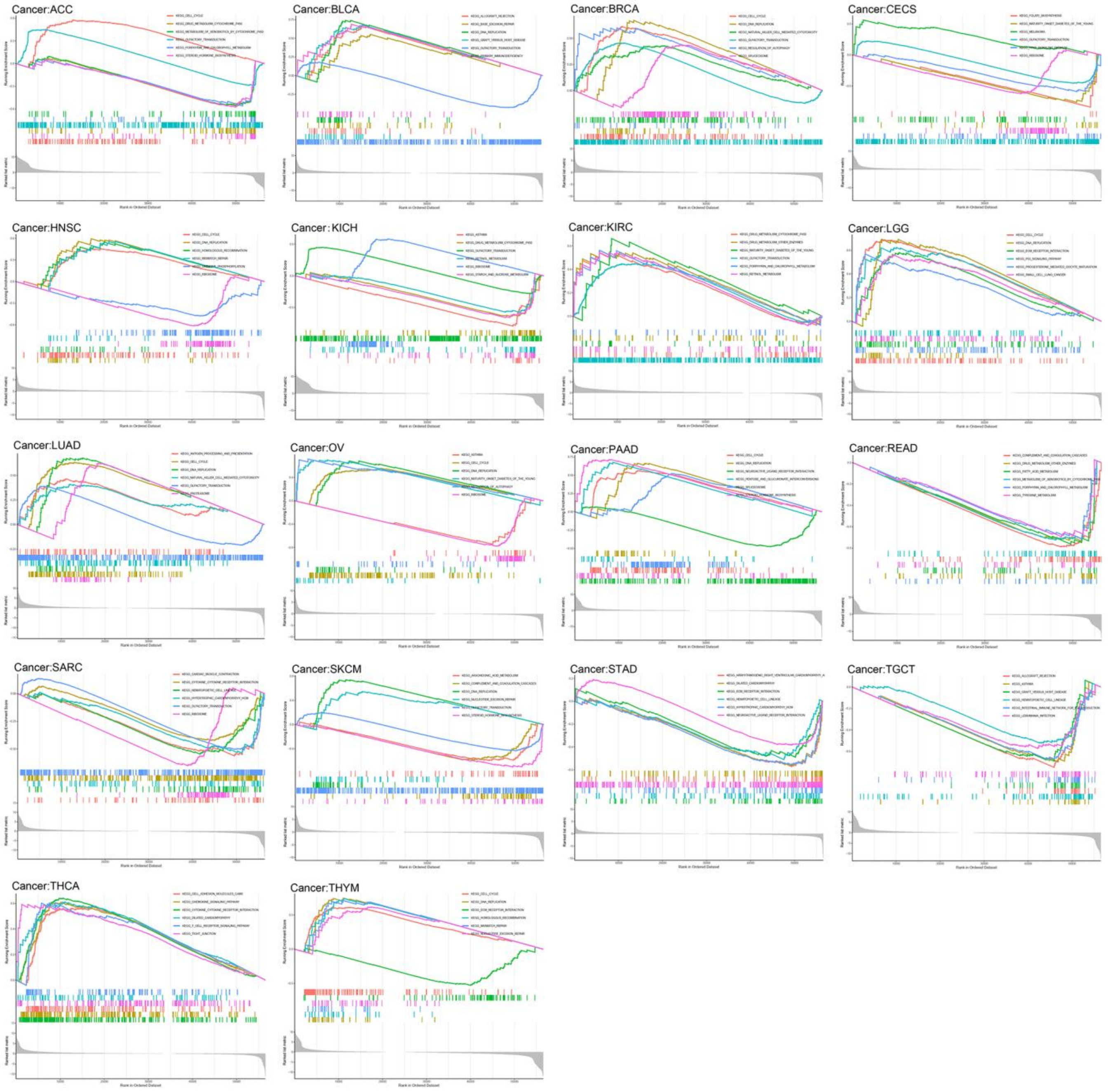

Supplementary Figure 4. Results of GSEA for FOXM1 correlation with signaling pathways in KEGG collection.
